# Modeling the prevalent germline *TP53* R337H mutation in mouse

**DOI:** 10.18632/oncotarget.26603

**Published:** 2019-01-18

**Authors:** Ji-Hoon Park, Ping-Yuan Wang, Paul M. Hwang

**Affiliations:** Cardiovascular Branch, DIR, NHLBI, National Institutes of Health, Bethesda, Maryland, USA

**Keywords:** Li-Fraumeni syndrome, p53 oligomerization, mouse model

The *TP53* gene encoded protein p53 plays a critical role in tumor suppression, while its germline mutation causes Li-Fraumeni syndrome (LFS), an autosomal dominant cancer predisposition disorder [1]. LFS is generally considered to be a rare inherited condition, but the unique founder mutation *TP53* R337H that gives rise to a variant of LFS is highly prevalent in southern and southeastern Brazil with an estimated mutation carrier frequency of ~0.3%, translating to hundreds of thousands of individuals [2]. Although its overall cancer penetrance is low compared with classic LFS mutations, *TP53* R337H has been associated with an increased risk of pediatric adrenocortical carcinoma relative to other cancers typically associated with LFS. The majority of LFS mutations occur within the DNA binding domain of p53 that compromises the transactivation of its target genes, but the R337H mutation is located in the oligomerization domain of the carboxy terminus with grossly preserved p53 transcriptional activity by *in vitro* studies [2]. However, because p53 functions as a tetramer with cooperative DNA binding properties, the putative pH-dependence of this mutant p53 for its oligomerization and DNA interaction may not be apparent by *in vitro* assays [2–4]. Given the high prevalence of this mutation and paucity of information on its characteristics *in vivo*, we recently reported the generation of a mouse model with knockin of the *p53* R334H mutation (human *TP53* R337H homolog) and examined how it affects tumorigenesis as well as the oligomerization and activity of p53 in response to DNA damage under physiological conditions [5].

**Figure 1:**
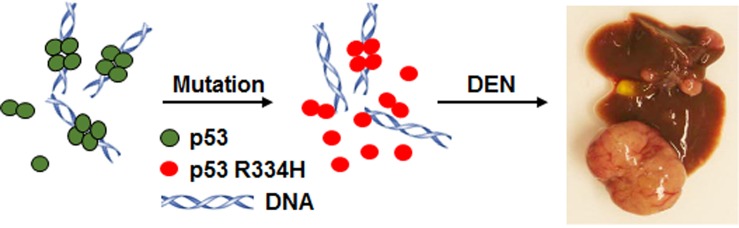
Mutant p53 R334H (red), mouse homolog of the human TP53 R337H mutation, forms less dimers and tetramers for DNA binding compared with wild-type p53 (green) in mouse liver after exposure to the liver carcinogen diethylnitrosamine (DEN) This is associated with decreased wild-type p53 transcriptional activity and increased DEN-induced liver tumor formation in the homozygous p53 R334H mutant mouse model.

Mice that are homozygous for the *p53* R334H mutation (*p53334H/H*) developed normally and did not show any significant difference in terms of either cancer incidence or life span compared with wild-type mice, consistent with the low cancer penetrance observed in humans with the *TP53* R337H mutation [5]. On the other hand, exposure to the carcinogen diethylnitrosamine (DEN) caused significantly increased liver tumor development in *p53334H/H* mice with more malignant histopathologic features. In parallel, there was evidence of increased DNA damage and decreased transactivation of p53 target genes in the DEN-treated liver tissue of *p53334H/H* mice compared with that of wild-type mice. Mechanistically, *p53334H/H* mouse liver tissue showed decreased levels of dimers and tetramers but increased monomers of p53 after DEN treatment, constituting the first *in vivo* demonstration of its diminished oligomerization capacity consistent with the observed decrease in its transcriptional activity (Figure 1).

Structural studies have suggested that arginine 337 within the α-helix motif of human p53 forms a salt bridge with aspartate 352 of another p53 molecule, helping to stabilize p53 homodimers that in turn dimerize to form tetramers [3, 4]. The substitution of arginine 337 to histidine, which has a lower pKa than arginine, has been proposed to disrupt the stabilization of these p53 homodimers due to the loss of protonation and salt bridge formation at physiologic pH [3]. Thus, the transcriptional activity of human p53 R337H may be more sensitive to intracellular pH than that of the wild-type protein. In this regard, it is notable that the adrenal gland, which has very active uptake and the highest concentration of ascorbic acid (vitamin C) in the human body [6], is susceptible to cancer development in carriers of the R337H mutation. Besides the adrenocortical carcinoma, another pediatric cancer observed in patients with the R337H mutation is carcinoma of the choroid plexus, which is involved in electrolyte transport and pH buffering of the cerebrospinal fluid [7]. This raises the possibility that drugs or conditions that alter intracellular pH could affect the oligomerization of mutant p53 R337H, thereby ameliorating its defective transcriptional activity *in vivo*. Because patients with the *TP53* R337H mutation have also been reported to have decreased plasma ascorbic acid levels [8], it is tempting to speculate that modulating tissue vitamin C levels or pH may alter endogenous p53 activity *in vivo* and affect tumorigenesis. Vitamin C deficiency is also common during pregnancy which could further potentiate the development of pediatric adrenocortical carcinoma in these genetically predisposed patients [9]. Finally, given the increasing interest in the use of metformin for chemoprevention in Li-Fraumeni syndrome [10], the increase in lactic acid production associated with the inhibition of respiration by metformin treatment could also be speculated to promote the tetramerization of p53 R337H for tumor suppression. With the availability of the *p53* R334H mouse model, some of these questions could be addressed in the laboratory and may lead to insights for follow up by clinical investigation with the goal of improving the management of this large population of patients with a unique p53 mutation.
